# Functional Integration Between the Two Brain Hemispheres: Evidence From the Homotopic Functional Connectivity Under Resting State

**DOI:** 10.3389/fnins.2020.00932

**Published:** 2020-10-02

**Authors:** Xinhu Jin, Xinyu Liang, Gaolang Gong

**Affiliations:** ^1^State Key Laboratory of Cognitive Neuroscience and Learning & IDG/McGovern Institute for Brain Research, Beijing Normal University, Beijing, China; ^2^Beijing Key Laboratory of Brain Imaging and Connectomics, Beijing Normal University, Beijing, China

**Keywords:** functional integration, left and right brain hemispheres, resting-state functional magnetic resonance imaging, resting-state functional connectivity, homotopic functional connectivity

## Abstract

Functional integration among neural units is one of the fundamental principles in brain organization that could be examined using resting-state functional connectivity (rs-FC). Interhemispheric functional integration plays a critical role in human cognition. Homotopic functional connectivity (HoFC) under resting state provide an avenue to investigate functional integration between the two brain hemispheres, which can improve the present understanding of how interhemispheric interactions affect cognitive processing. In this review, we summarize the progress of HoFC studies under resting state and highlight how these findings have enhanced our understanding of interhemispheric functional organization of the human brain. Future studies are encouraged to address particular methodological issues and to further ascertain behavioral correlates, brain disease’s modulation, task influence, and genetic basis of HoFC.

## Introduction

Brain activity globally interacts at multiple levels with functional integration ranging from neural units to brain regions to overall behavioral output, leading to the emergence of widespread patterns of correlations among neuronal groups (Tononi et al., 1994). How the left and right hemispheres are functionally integrated is a crucial issue in neuroscience. For the two hemispheres, functional integration refers to the interaction between specialized regions, enabling interhemispheric long-range synchronization and information flow (Friston, 2004; van den Heuvel and Sporns, 2013). Such functional integration has been found to play a vital role in cognitive processing, such as visuospatial attention, speech perception and creative thinking (Gotts et al., 2013; Nuttall et al., 2018; Chen et al., 2019).

Various neuroimaging techniques have been applied to investigate functional integration between the two brain hemispheres. For example, Horwitz et al. (1984) used regional metabolic rates acquired by positron emission tomography (PET) to characterize the functional integration among different brain regions. They found that all regions were significantly correlated with their contralateral homologs. Likewise, Duffy et al. (1996) inspected interhemispheric functional integration via resting-state electroencephalogram (EEG) recordings and revealed synchronized electrical activity between the left and right hemispheres. With the rapid development of functional magnetic resonance imaging (fMRI) in recent years, resting-state fMRI (rs-fMRI) has been employed to explore interhemispheric functional integration more frequently, given its advantages such as relatively high spatial and temporal resolution without any invasion over other neuroimaging techniques (Biswal et al., 1995). The rs-fMRI provides a powerful method to investigate functional integration between different brain regions by calculating resting-state functional connectivity (rs-FC) that is deemed to reflect the interactions across different cerebral regions (Fox and Raichle, 2007). By means of the interhemispheric rs-FC, we could directly quantify functional integration between the two brain hemispheres and thus have a great chance to determine how interhemispheric functional integration affects cognitive processing (Gee et al., 2011).

rs-FC analyses to detect patterns of functional integration between the two brain hemispheres were firstly applied in the motor system (Biswal et al., 1995). Since then, rs-fMRI studies have revealed whole-brain patterns of synchronized spontaneous activity between homotopic regions in left and right hemispheres, which is referred to as homotopic functional connectivity (HoFC) (Salvador et al., 2005; Stark et al., 2008). As demonstrated, HoFC is a robust feature with a peculiar interior distribution corresponding to the functional hierarchy, which does not follow the general law defined by the spatial distance (Salvador et al., 2005; Stark et al., 2008; Tobyne et al., 2016). Meanwhile, it has been found to correlate with the performance of cognitive tasks, such as visuospatial attention (Gracia-Tabuenca et al., 2018) and executive function (Zhao et al., 2020). As summarized in Table 1, a number of studies have achieved progress in functional integration between the two brain hemispheres by virtue of HoFC measurements under resting state.

**TABLE 1:** Basic information for published rs-fMRI studies of HoFC.

**Author (years)**	**Modeling method**	**Scale**	**Scope**	**Main findings**
Quigley et al. (2003)	Volume-based	Voxel-level	Seed regions	C
Salvador et al. (2005)	Volume-based	ROI-level	Whole brain	A
Stark et al. (2008)	Volume-based	ROI-level	Whole brain	A
Johnston et al. (2008)	Volume-based	ROI-level	Seed regions	C
Zuo et al. (2010)	Volume-based	Voxel-level	Whole brain	A/B
Smyser et al. (2010)	Volume-based	ROI-level	Seed regions	B
Kelly et al. (2011)	Volume-based	Voxel-level	Whole brain	E
Hoptman et al. (2012)	Volume-based	Voxel-level	Whole brain	E
Owen et al. (2013)	Volume-based	ROI-level	Seed regions	C
Guo et al. (2013)	Volume-based	Voxel-level	Whole brain	E
Yang et al. (2014)	Volume-based	Voxel-level	Whole brain	E
Shen et al. (2015)	Volume-based	ROI-level	Whole brain	A
Tzourio-Mazoyer et al. (2016)	Volume-based	ROI-level	Whole brain	B/F
Ren et al. (2015)	Volume-based	Voxel-level	Whole brain	E
Luo et al. (2015)	Volume-based	Voxel-level	Whole brain	E
Tobyne et al. (2016)	Surface-based	Vertex-level	Whole brain	A
Emerson et al. (2016)	Volume-based	ROI-level	Seed regions	B
Hermesdorf et al. (2016)	Volume-based	Voxel-level	Whole brain	E
Roland et al. (2017)	Volume-based	ROI-level/voxel-level	Seed regions/whole brain	C
Guo et al. (2018)	Volume-based	Voxel-level	Whole brain	E
Gracia-Tabuenca et al. (2018)	Volume-based	Voxel-level	Whole brain	B/D/F
Mollink et al. (2019)	Volume-based	ROI-level	Whole brain	C
Fan et al. (2018)	Volume-based	Voxel-level	Whole brain	E
Luo et al. (2018)	Volume-based	Voxel-level	Whole brain	E
Zhao et al. (2020)	Volume-based	Voxel-level	Whole brain	B/D
Chen et al. (2020)	Volume-based	Voxel-level	Whole brain	E
Ye et al. (2020)	Volume-based	Voxel-level	Whole brain	E

*rs-fMRI: resting-state functional magnetic resonance imaging; HoFC: homotopic functional connectivity; ROI: region of interest. The letters in the main findings column are denoted as follows: A, basic characteristics of HoFC; B, physiological effects on HoFC; C, structural foundation of HoFC; D, correlation between HoFC and cognition; E, HoFC abnormalities in brain diseases; F, the role of HoFC in functional lateralization.*

This review aims to summarize the studies of functional integration between the two brain hemispheres by using HoFC under resting state and to highlight how the findings have enhanced our understanding of hemispheric functional organization in the human brain. For the summary of existing literatures, we included six sections: (1) Methods of Calculating HoFC, (2) Physiological Effects on HoFC, (3) Structural Foundation of HoFC, (4) Correlation Between HoFC and Cognition, (5) HoFC Abnormalities in Brain Diseases, and (6) the Role of HoFC in Functional Lateralization. Future considerations are elaborated into four topics: (1) Lifespan Trajectory of HoFC, (2) Genetic Basis of HoFC, (3) HoFC Under Tasking State, and (4) the Functional Meaning of HoFC to Cognition in Healthy People.

## HoFC and Interhemispheric Functional Integration

In this part, we review relevant rs-fMRI findings of functional integration between the two brain hemispheres by using HoFC.

### Methods of Calculating HoFC

**FIGURE 1:**
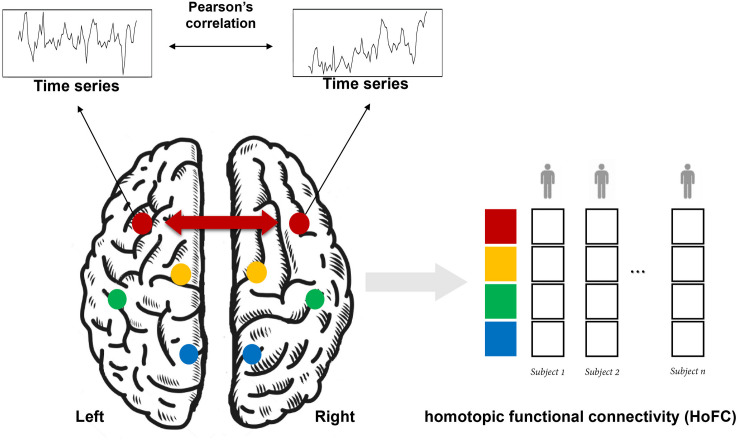
A schematic flowchart of the definition of homotopic functional connectivity (HoFC). The homotopic pair could be mirrored voxels or vertices or from an atlas with defined homotopic regions. For a given homotopic pair, interhemispheric HoFC is calculated as the Pearson’s correlation between the time series of this location and its homotopic contralateral counterpart. Finally, a whole-brain vector of HoFC is obtained for each subject.

As shown in Figure 1, HoFC is calculated by Pearson’s correlation between the time series of homotopic locations under resting state, which is the most important way to reflect functional integration between the two brain hemispheres. In early stages, homotopic locations are defined as corresponding anatomical areas or spatial locations in opposite hemispheres (Salvador et al., 2005; Stark et al., 2008; Zuo et al., 2010). Recently, researchers proposed that each brain region has a unique homotopic contralateral counterpart with a maximal rs-FC (Joliot et al., 2015). Specifically, HoFC is computed by using different methodological definitions with various spatial scales, such as regions of interest (ROIs), voxel-level in volume, or vertex-level on cortical surface. Below, we summarized different methods to obtain HoFC.

#### Atlas-Based HoFC

**FIGURE 2:**
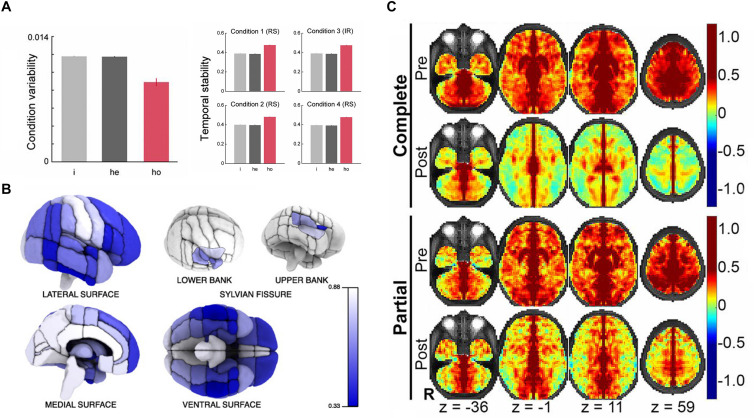
The basic characteristics and structural foundation of HoFC. **(A)** The condition variability and temporal stability for different types of functional connectivity, including intrahemispheric (i), heterotopic (he), and homotopic (ho). The figure is adapted from Shen et al. (2015). Permission to reuse Figures 2, 3 from the article was granted by PNAS. **(B)** Mean interhemispheric HoFC indicated for all cortical regions. The figure is adapted from Stark et al. (2008). Permission to reuse Figure 4 from the article was granted by Society for Neuroscience (“Copyright [2008] Society for Neuroscience”). **(C)** HoFC computed as the Fisher Z-transformed VMHC before (first and third rows) and after (second and forth rows) corpus callosotomy (complete and partial). The figure is adapted from Roland et al. (2017). Permission to reuse Figure 4 from the article was granted by PNAS.

The majority of early studies adopted anatomical parcellations to define homotopic locations in the left and right hemispheres. Initially, rs-fMRI studies mostly concentrated on a specific cortical or subcortical brain region and have observed a high level of interhemispheric correlation in those regions (Biswal et al., 1995; Lowe et al., 1998; Cordes et al., 2000). Until 2005, Salvador et al. (2005) first noticed that synchronous activity across homotopic regions was a ubiquitous phenomenon among the whole brain. This first whole-brain analysis based on human rs-fMRI data calculated the HoFC via the automated anatomical labelling (AAL) atlas (Salvador et al., 2005), which is proposed by Tzourio-Mazoyer et al. (2002) and comprises 45 corresponding anatomical ROIs in each cerebral hemisphere. Based on AAL atlas as well, Shen et al. (2015) built on their work under resting state and found HoFC was significantly higher than heterotopic or intrahemispheric FC, validated in both human and macaque datasets (Shen et al., 2015). In addition, they discovered HoFC was more stable and resistant across both varied conditions and temporal changes than heterotopic or intrahemispheric FC (Figure 2A). In 2008, Stark et al. (2008) used the Harvard–Oxford Structural Atlas to divide the entire cerebrum into 112 homotopic regions, 56 in each brain hemisphere. They reported a pattern of regional variation in temporally correlated low-frequency brain activity between the two hemispheres, suggesting that interhemispheric coordination may vary across homotopic regions in the human brain. As shown in Figure 2B, despite interhemispheric HoFC across the whole brain regions was robust, higher HoFC value was more exhibited in primary sensorimotor cortices than in higher-order association areas. In recent years, studies have suggested that it would be more reasonable and advantageous for the investigation of cerebral functional organization by using homotopic ROIs derived from functional parcellation (Joliot et al., 2015; Tzourio-Mazoyer et al., 2016). Therefore, Joliot et al. (2015) developed a functional parcellation based on the intrinsic connectivity of homotopic areas, namely the Atlas of Intrinsic Connectivity of Homotopic Areas (AICHA). This atlas covers the entire cerebral gray matter and includes 192 homotopic region pairs, which has already been used in studies related to HoFC (Tzourio-Mazoyer et al., 2016).

#### Voxel-Based HoFC

In order to investigate the HoFC on a finer scale, Zuo et al. (2010) provided a comprehensive examination of HoFC by using a voxel-wise measure called voxel-mirrored homotopic connectivity (VMHC). Specifically, the individual rs-fMRI data were registered to a symmetrical brain template in Montreal Neurological Institute (MNI) space, which was generated by averaging the group-specific mean normalized T1 image and its left–right mirrored image. This registration step roughly ensures each voxel in one hemisphere has a homotopic voxel in contralateral hemisphere. Then, the Pearson’s correlation coefficient between the resting-state time series of each voxel and its symmetrical interhemispheric counterpart was calculated as the VMHC. By using this method, they also verified consistent regional variation of HoFC with Stark et al. (2008). In contrast to the atlas-based HoFC, the VMHC approach allows a greater and finer appreciation of regional variation within larger cortical structures, such as the cingulate, precentral and postcentral gyri (Zuo et al., 2010).

#### Surface-Based HoFC

To achieve a better alignment of homologous location between the two brain hemispheres, a surface-based HoFC can be used (Jo et al., 2012).

Using the landmark-based anatomical correspondence, Jo et al. (2012) first putatively established vertex-wise homotopic locations between the two hemispheres on a non-symmetric template brain. They identified the improved precision of this surface-based approach over the previous volume-based flipping method. Following this study, Tobyne et al. (2016) further introduced a computationally efficient technique, surface-based HoFC, that leveraged surface-based registration to improve the spatial specificity and accuracy of cortical HoFC under resting state. The surface-based HoFC for each pair of homologous vertices was calculated by using the pairwise Pearson’s correlation between the extracted time series. This technique is presented as a sensitive measure of HoFC in the human brain and is proved to be reliable both within and across subjects (Tobyne et al., 2016). Particularly, this method presents a more constrained pattern of HoFC along the medial surface. As noted, either the volumetric smoothing or greater accuracy of spherical registration techniques might lead to discrepancies between volume-based and surface-based HoFC (Tobyne et al., 2016).

### Physiological Effects on HoFC

**FIGURE 3:**
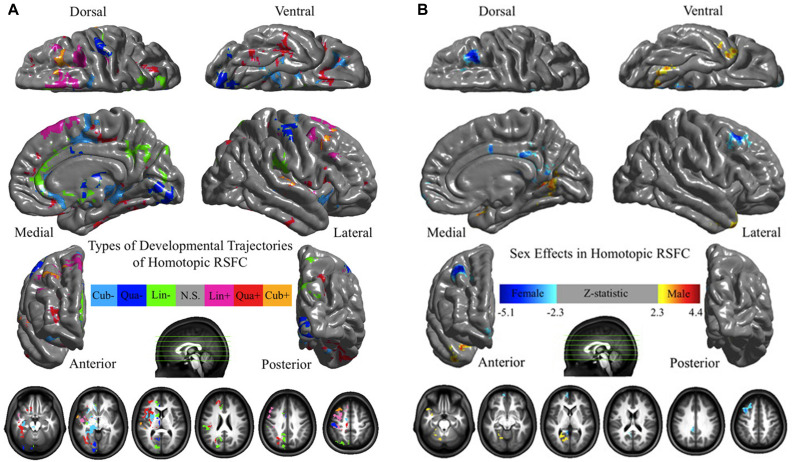
The physiological effects on HoFC. **(A)** Age effects on HoFC. The linear (Lin), quadratic (Qua), and cubic (Cub) effects of age on VMHC were shown at each voxel, evaluating through multiple linear regressions model. **(B)** Sex effects of HoFC. One-sample tests on regression coefficients of sex was performed for both positive and negative contrasts. These figures are adapted from Zuo et al. (2010). Permission to reuse Figures 3, 5 from the article was granted by the Creative Commons Attribution-Non-commercial-Share Alike 3.0 Unported License (CC-BY-NC-SA).

The human brain is affected by a number of physiological factors, such as age and sex. Zuo et al. (2010) systematically investigated age-related HoFC changes under resting state by using VMHC in 214 healthy individuals (age range: 7–85 years). As shown in Figure 3A, sensorimotor regions displayed a HoFC increase, whereas higher-order association regions showed a HoFC decrease along with age. In addition, they detected complex maturational curves, such as the quadratic trajectories of insula and lingual gyrus as well as the cubic trajectories of putamen and superior frontal gyrus. Furthermore, sex-related differences in HoFC between males and females were discovered, with males having weaker HoFC in the medial and dorsal regions as well as greater HoFC in the parahippocampal and fusiform gyri (Figure 3B). Moreover, there were interactions between age and sex on resting-state HoFC in the dorsolateral prefrontal cortex and amygdala. In a recent study, Zhao et al. (2020) examined VMHC in 93 healthy volunteers between 19 and 85 years of age and observed reductions in VMHC of the ventromedial prefrontal cortex, inferior parietal lobule, dorsal anterior cingulate cortex, hippocampus and insula along with age. In addition to these VMHC approaches, Tzourio-Mazoyer et al. (2016) used the functionally driven homotopic atlas (AICHA) to define the homotopic regions of the human brain. They recruited 291 individuals (age range: 18–57 years; 148 men, 143 women) and found that mean HoFC (averaged across the 36 pairs of lateral homotopic regions) significantly decreased with age and was larger in males than females. Together, HoFC in lifespan indicated a global decrease related with age (Zuo et al., 2010; Tzourio-Mazoyer et al., 2016). In specific regions, Zhao’s study found negative correlation, while Zuo’s study reported various development trajectories (Zuo et al., 2010; Zhao et al., 2020). The discrepancies might be due to different statistical models in their studies, with a simple GLM in Zhao’s work and a more complicated one including both quadratic and cubic terms in Zuo’s work. In Zuo et al. (2010), the age distribution is skewed, which could affect the results of relationship between age and HoFC. Unlike the three studies above, Gracia-Tabuenca et al. (2018) focused on typically developing children ranging in age from 6 to 10 years. In a sample of 60 participants, significant sex effects were noted in the frontal pole, but no age effects were identified.

In infant group, Emerson et al. (2016) tried to portray the development curve of interhemispheric functional asymmetry during the first 2 years of life, by leveraging a large sample of human infants with longitudinal rs-fMRI scans. The interhemispheric HoFC exhibited three predominant patterns, including one trajectory for sensory regions, one trajectory for higher-order association regions and another trajectory for language-related regions. Concentrated on an earlier stages from 26 weeks postmenstrual age (PMA), Smyser et al. (2010) obtained resting-state networks based on rs-FC data of preterm infants, and they found the networks with more mature forms in older infants consisted of localized interhemispheric HoFC between homotopic counterparts, demonstrating a regionally age-specific development pattern.

### Structural Foundation of HoFC

Like other rs-FC, HoFC is likely constrained by structural connections of the brain (Honey et al., 2009). Anatomically, the corpus callosum (CC) is the major neural pathway that connects the homotopic regions of the left and right hemispheres (Innocenti, 1986) and putatively plays a pivotal role in mediating HoFC under resting state (Tzourio-Mazoyer et al., 2016; Gracia-Tabuenca et al., 2018).

This line of research was explored mainly by using patients with CC defects. In 2003, Quigley et al. (2003) found that patients with agenesis of the corpus callosum (AgCC) showed diminished interhemispheric functional connectivity in the motor and auditory cortices compared with healthy participants. Owen et al. (2013) extracted 17 resting-state networks of patients with AgCC (including partial and complete) and well-matched healthy controls. They detected a striking loss of interhemispheric HoFC of the insular-opercular regions, posterior cingulate cortex, and precuneus in AgCC subjects. Johnston et al. (2008) presented a rs-FC study of a child patient with medically refractory epilepsy both before and after complete section of the CC, which demonstrated reduced interhemispheric HoFC and preserved intrahemispheric connectivity postoperatively. Further, Roland et al. (2017) evaluated rs-FC in 22 patients with intractable epilepsy after surgical section of the CC; they found markedly reduced interhemispheric HoFC in the human brain, which was more profound in multimodal associative areas than in primary regions. The existence of polysynaptic FC between distant cerebral regions was inferred by comparison of partial vs. complete callosotomy (Figure 2C). These results together indicated that callosal anatomical connections played a critical role in the existence and maintenance of interhemispheric HoFC, which provided crucial insights into general relationship between structural connection and functional connectivity in the human brain. These approaches, however, were unable to characterize interindividual differences of healthy people because of the particularity of subjects with CC defects, although they did have relatively high biological specificity and interpretability.

A large number of studies have applied diffusion magnetic resonance imaging (dMRI) to study structural connections of the human brain non-invasively. Using dMRI and rs-fMRI data in 11354 subjects from the UK Biobank, Mollink et al. (2019) focused on interhemispheric connectivity and corresponding commissural fibers passing through the CC between pairs of homotopic cerebral regions. They constructed multivariate models to link interhemispheric structural connectivity and functional connectivity, showing that ninety percent of human brain regions were statistically related between HoFC under resting state and white matter microstructure of CC.

In summary, the results both in patients and healthy people provide strong evidence supporting that corticocortical CC connectivity serves as pivotal structural basis underlying the resting-state HoFC between homotopic cortical regions of the two hemispheres.

### Correlation Between HoFC and Cognition

As an important representation of functional integration between the two brain hemispheres, HoFC under resting state was found to be significantly correlated with human’s cognitive activities, such as visuospatial attention and executive function. Gracia-Tabuenca et al. (2018) discovered that the VMHC of the occipital fusiform gyrus and the lingual gyrus was negatively associated with the performance in visual long-term memory tasks. In addition, Zhao’s study found that the VMHC of the ventromedial prefrontal cortex and insula showed a negative linear relationship with the performance on the color-word inhibition task mainly about executive function (Zhao et al., 2020).

### HoFC Abnormalities in Brain Diseases

A number of studies have made direct comparisons of HoFC between different patient groups and healthy controls, most of which utilized VMHC to represent HoFC.

Compared to healthy controls, some studies have demonstrated increased HoFC in patient groups, such as idiopathic generalized epilepsy (Yang et al., 2014) and paroxysmal kinesigenic dyskinesia (Ren et al., 2015), while others reported decreased HoFC, ranging from cocaine addiction (Kelly et al., 2011), schizophrenia (Hoptman et al., 2012), Parkinson’s disease (Luo et al., 2015), major depressive disorder (MDD) (Hermesdorf et al., 2016), to amnestic mild cognitive impairment (Luo et al., 2018). Some brain diseases, including HBV-related cirrhosis (Ye et al., 2020) and subacute stroke (Chen et al., 2020), showed both increases and decreases of HoFC in different brain regions. Although majority of results are consistent across studies, discrepancies exist. For example, several studies have used VMHC under resting state to explore the difference between MDD patients and healthy people (Guo et al., 2013, 2018; Hermesdorf et al., 2016; Fan et al., 2018). Compared to healthy controls, most studies found no VMHC increase and a significant VMHC decrease in the cuneus, posterior cingulate cortex and precuneus in MDD patients. However, Hermesdorf et al. (2016) reported a VMHC decrease in the putamen, superior temporal gyrus and insula, which exhibited a wider brain region change in the VMHC in patients with MDD than other studies. These discrepancies might be due to the differences among studies in inclusion criteria and the number of participants, fMRI acquisition, analysis methods, and so on. Regarding the relationship with behavior and symptoms, the HoFC under resting state in specific brain regions was found to be associated with scores of disease-related scales in patient groups. For instance, Kelly et al. (2011) revealed a correlation between HoFC within the regions in the dorsal attention network and self-reported lapses of attention in the cocaine-dependent group. For schizophrenia, Hoptman et al. (2012) showed that HoFC in the postcentral gyrus extending into the precentral gyrus was associated with Positive and Negative Syndrome Scale (PANSS) total scores.

### The Role of HoFC in Functional Lateralization

In addition to the functional integration between the two brain hemispheres, another key organization principle of the human brain is functional lateralization. Functional lateralization is acquired by comparing the brain activity patterns between a region in one hemisphere and its contralateral homotopic counterpart in the other (Tomasi and Volkow, 2012; Nielsen et al., 2013). Previous studies have reported that HoFC is related to functional lateralization. Tzourio-Mazoyer et al. (2016) investigated this relationship in 297 healthy volunteers, including 153 left-handers (LHs). They found a significant negative correlation between resting-state HoFC and language task-induced functional lateralization, suggesting a translation from enhanced interhemispheric cooperation in the resting state into increased interhemispheric cooperation during the language production. In addition, all the subjects in this study were classified into three different types according to functional lateralization of language task: typicals, strong-atypicals (only LHs) and ambilaterals. A handedness effect on HoFC was also discovered by the lateralization subgroup comparison, with left-handed ambilaterals having significantly greater HoFC than the other groups. Gracia-Tabuenca et al. (2018) also examined these two brain functional properties, both defined based on rs-FC. Their results identified that HoFC was strongly negatively correlated with functional lateralization across the whole human brain.

Combined with previous findings of the fundamental role of CC, HoFC under resting state might reflect the effect of CC on functional lateralization (Tzourio-Mazoyer et al., 2016). In fact, many studies have demonstrated the excitatory role of CC in interhemispheric processing (Galaburda et al., 1985, 1990). The CC may enforce cerebral processing integration between the left and right hemispheres (Lezak et al., 2004) and activate the unstimulated hemisphere (Yazgan et al., 1995). According to this hypothesis, more symmetric brains have stronger interhemispheric connections, indicating a correlation between a smaller CC and greater lateralization (Bloom and Hynd, 2005). The significant negative correlations between HoFC and functional lateralization in studies above (Tzourio-Mazoyer et al., 2016; Gracia-Tabuenca et al., 2018) therefore provided supporting evidence for the excitatory role of the CC in interhemispheric processing in the human brain.

## Future Considerations

We will elaborate several specific aspects about HoFC in the following sections, which deserve particular consideration in future studies.

### Lifespan Trajectory of HoFC

By means of a range of neuroimaging methods, complex changes in the structure and function of the human brain across the lifespan are becoming accessible for analysis (Cao et al., 2014). As early as the fetal period during the second half of gestation, many human brain networks, involving both corticocortical and thalamocortical connections, are initially appeared and established (Kostović and Jovanov-Milošević, 2006). Findings from infants using HoFC under resting state suggested that the developing human brain might increase its interhemispheric functional symmetry to govern multiple functional systems, which might be a general principle in the first year of life (Smyser et al., 2010; Emerson et al., 2016). Therefore, the exploration of functional integration between the two brain hemispheres should also start at earlier stages of brain development such as the fetal or neonatal stage. In most existing lifespan HoFC studies, the age range of subjects were disequilibrium and mostly located at young adults group (Zuo et al., 2010; Tzourio-Mazoyer et al., 2016; Zhao et al., 2020). In future studies, it is warranted to map out lifespan trajectories of HoFC for different homologous regions, using a uniformly distributed lifespan cross-sectional or longitudinal data.

### Genetic Basis of HoFC

Many studies have shown that resting-state FC and networks are considerably heritable, suggesting that HoFC might be influenced by genetic factors (Glahn et al., 2010; Ge et al., 2017). As the structural foundation of HoFC between the two brain hemispheres, the CC is found highly heritable as well. For example, previous studies have confirmed both the size and microstructure of the CC are strongly controlled by genetic factors (Kanchibhotla et al., 2014; Woldehawariat et al., 2014; Vuoksimaa et al., 2017). To date, it remains unexplored whether and how genetic factors influence HoFC. In the future, it would be interesting to investigate the heritability of HoFC at multiple levels by employing advanced imaging genetics techniques, such as twin study with ACE model, genome-wide association study (GWAS) or gene set enrichment analysis (GSEA). The findings would help to understand the relationship between interhemispheric functional integration and genetic factors, as well as environmental factors.

### HoFC Under Tasking State

Compared to rs-fMRI, tasking-state fMRI (ts-fMRI) has been largely used to identify functional activities evoked by specific cognitive tasks. Measurements of brain activity actually reflect a mixture of spontaneous and evoked activities during a specific task. Recently, more and more studies started focusing on tasking-state functional connectivity (ts-FC). Many functional organization properties of the human brain under resting state have been recognized during the tasking state. Across dozens of different tasks, Cole et al. (2014) identified a whole-brain network architecture, which was highly similar to the one presented in resting state. In addition, there was a small but consistent group of changes across different tasks, suggesting the existence of a task-general network architecture distinguished from the resting state. These results indicated that the functional architecture of the human brain under tasking state was likely composed of an intrinsic architecture that appeared under resting state and secondarily shaped by evoked task-general and task-specific changes. In the future, the similarities and differences of HoFC between the resting state and tasking state can be thoroughly explored, which may enhance the understanding about the relationship between the two functional states of the human brain, from an interhemispheric functional integration perspective.

### The Functional Meaning of HoFC to Cognition in Healthy People

To better understand cognitive consequence of functional integration between the two brain hemispheres, more studies are encouraged to explore the functional meaning of HoFC in human brain. As reviewed above, HoFC has been found to be related with particular cognitions, behaviors and specific symptom in both healthy and diseases. In healthy people, studies revealed only a few cognitions related with regional HoFC under resting state, such as executive function, visuospatial attention, language, etc. The association with cognition was more frequently reported in brain disease than healthy people, which might due to the limited individual difference of HoFC in healthy people.

Currently, some available public data, such as the Human Connectome Project (HCP) or UK biobank, provide both neuroimaging and behavior data in a large sample of normal people, allowing for thorough investigation of brain-cognition relationships with a strong statistical power. More studies are encouraged to directly investigate the HoFC-cognition associations in healthy people. By studying these large sample datasets alone or combining with them, researchers may capture subtle associations between the HoFC and complex cognitions/behaviors. Advanced analysis techniques are encouraged for the investigation of HoFC-cognition relationship as well, such as multivariable correlation or machine learning.

## Conclusion

This article reviewed existing studies of HoFC under resting state that reflects to some degree functional integration between the two brain hemispheres. Future studies are encouraged to better characterize lifespan trajectories, genetic basis, tasking-state modulation, cognitive correlates of HoFC.

## Author Contributions

XJ, XL, and GG contributed to the conception and design of the review. XJ and XL wrote the first draft of the manuscript. All authors contributed to manuscript revision and approved it for publication.

## Conflict of Interest

The authors declare that the research was conducted in the absence of any commercial or financial relationships that could be construed as a potential conflict of interest.
